# NADPH Oxidase-Dependent Superoxide Production in Plant Reproductive Tissues

**DOI:** 10.3389/fpls.2016.00359

**Published:** 2016-03-31

**Authors:** María J. Jiménez-Quesada, José Á. Traverso, Juan de Dios Alché

**Affiliations:** ^1^Plant Reproductive Biology Laboratory, Department of Biochemistry, Cell and Molecular Biology of Plants, Estación Experimental del Zaidín, Spanish National Research Council (CSIC)Granada, Spain; ^2^Department of Cell Biology, Faculty of Sciences, University of GranadaGranada, Spain

**Keywords:** NADPH oxidase, NOX, pollen, pistil, pollen–pistil interaction, Rboh, sexual plant reproduction

## Abstract

In the life cycle of a flowering plant, the male gametophyte (pollen grain) produced in the anther reaches the stigmatic surface and initiates the pollen–pistil interaction, an important step in plant reproduction, which ultimately leads to the delivery of two sperm cells to the female gametophyte (embryo sac) inside the ovule. The pollen tube undergoes a strictly apical expansion characterized by a high growth rate, whose targeting should be tightly regulated. A continuous exchange of signals therefore takes place between the haploid pollen and diploid tissue of the pistil until fertilization. In compatible interactions, theses processes result in double fertilization to form a zygote (2n) and the triploid endosperm. Among the large number of signaling mechanisms involved, the redox network appears to be particularly important. Respiratory burst oxidase homologs (Rbohs) are superoxide-producing enzymes involved in a broad range of processes in plant physiology. In this study, we review the latest findings on understanding Rboh activity in sexual plant reproduction, with a particular focus on the male gametophyte from the anther development stages to the crowning point of fertilization. Rboh isoforms have been identified in both the male and female gametophyte and have proven to be tightly regulated. Their role at crucial points such as proper growth of pollen tube, self-incompatibility response and eventual fertilization is discussed.

## Superoxide Generation in Plants

The superoxide radical ($O_{2}^{•-}$), a short-lived reactive oxygen specie (ROS) characterized by moderate reactivity, is able to trigger a cascade of reactions (enzymatic, metal-catalyzed, and even direct reactions) in order to produce others ROS species. $O_{2}^{•-}$ is produced in chloroplasts, mitochondria, the endoplasmic reticulum, and peroxisomes due to their normal metabolism (Sharma et al., 2012).

Apart from the production of free radicals that occurs as a result of the side reactions of metabolism and electron leakage, the plant oxidative burst was first described as the physiologically-controlled and rapid ROS generation during the early responses to pathogen infections, similarly to what occurs in animal phagocytic cells. This process has been described as involving the activity of NADPH oxidase enzymes, called Rbohs, in plants (Lamb and Dixon, 1997). Although the Rboh family seems to be the major source of the generated ROS as described above, other systems, including cell wall-bound peroxidase (POXs) (Bolwell et al., 2002), oxalate oxidase (Hu et al., 2003), amine oxidase (Angelini and Federico, 1989), and quinone reductase (Schopfer et al., 2008), have been proposed. With regard to plant POXs, $O_{2}^{•-}$ generation occurs via the H_2_O_2_-dependent POX cycle as well as via the H_2_O_2_-independent oxygenation cycle (Kimura et al., 2014). An apoplastic peroxidase-catalyzed oxidative burst following biotic stress has been described in different species such as *Arabidopsis thaliana* or *Phaseolus vulgaris* (Bolwell et al., 2002; O’Brien et al., 2012), although NADPH oxidase and other sources including mitochondria also contribute to ROS generation (O’Brien et al., 2012). On the other hand, the oxidative response induced by hypo-osmolarity in cell cultures has been shown to originate from NADPH oxidase activity in tobacco, whereas, a cell wall-POX capable of producing superoxide, has been identified in *Arabidopsis* (Rouet et al., 2006). In a different study dealing with intact and excised sunflower roots, distinct sources of superoxide were detected: extracellular POX appeared in both samples, while NADPH oxidase was present in intact roots only (Garrido et al., 2012). The studies mentioned above analyze the relative contributions of peroxidases and NADPH oxidases to ROS production by using inhibitors which affect enzymes differently (Bindschedler et al., 2006; Daudi et al., 2012). Thus, the effect of cytochrome inhibitors potassium cyanide and sodium azide, was compared with the impact caused by diphenyleneiodonium (DPI), which is capabable of inhibiting Rboh activity by affecting dimerization and mobility (Hao et al., 2014). Likewise, the ability of Cd^2+^ ions to inhibit NADPH oxidase was used to distinguish between NADPH oxidase -produced $O_{2}^{•-}$ and the superoxide derived from mitochondria (Heyno et al., 2008).

## NADPH Oxidase-Dependent Oxidative Burst: A Plant Overview

NADPH oxidase enzymes belong to a family of transmembrane proteins able to transport electrons across a membrane -usually the plasma membrane- from a cytosolic electron donor to oxygen -the extracellular acceptor- thus catalyzing the generation of $O_{2}^{•-}$ (Lambeth, 2004). However, the lifespan of superoxide molecules, which are rapidly dismutated to H_2_O_2_ either spontaneously or through the action of apoplastic superoxide dismutases (SOD), is extremely short (Bowler et al., 1994). NADPH oxidase (NOX) activity was first described in mammal phagocytic cells and consists of two plasma membrane proteins, gp91phox and p22phox (phox for phagocyte oxidase). This protein complex is regulated by its interaction with other cytosolic proteins (p47phox, p67phox, p40phox, and Rac2) producing the active form (Cross and Segal, 2004; Lambeth, 2004). Although these mammalian protein complexes are the most studied NOXs, these enzymes have also been characterized in other animals, fungi, and plants. NADPH oxidase enzymes share a basic structure consisting of six transmembrane domains, two heme-binding sites and a long cytoplasmic C-terminal which includes FAD and NADPH binding sites (Bedard et al., 2007).

In plants, the *Rboh* gene was first isolated in *Oryza sativa* as a homolog of the *gp91phox* mammal gene, which was later described in tomato, *Arabidopsis*, potato, and tobacco (Groom et al., 1996; Keller et al., 1998; Torres et al., 1998; Amicucci et al., 1999; Simon-Plas et al., 2002; Yoshioka et al., 2003). The *Arabidopsis* genome encodes at least 10 Rboh homologues, which usually show different gene expression patterns both in different plant organs and at different developmental stages, suggesting that these isoforms have no overlapping function (Torres et al., 1998; Dangl and Jones, 2001). Nevertheless, *RbohD* appears to be highly expressed in the whole plant and probably acts as a housekeeping gene (Hao et al., 2014). Rbohs were initially described as plasma membrane proteins (Sagi and Fluhr, 2001; Takeda et al., 2008) associated with specific plasma membrane microdomains, and with sterols being regarded as crucial for a proper localization (Liu et al., 2009; Posé et al., 2009). In fact, their distribution, which is not uniform, is characterized by discrete dynamic spots with a highly heterogeneous diffusion coefficient (Hao et al., 2014). In the same study, the effective amount of the isoform analyzed (RbohD) was shown to be regulated by both endocytosis and transport to the vacuole for degradation. Rboh accumulation has actually been detected not only in the plasma membrane but is also associated with internal endomembranes (Heyno et al., 2008; Drerup et al., 2013). According to these findings, Rboh-dependent ROS were also detected in vesicles in response to salt stress and during ABA-induced stomatal closure (Leshem et al., 2007, 2010). Although these locations could initially be considered to be surprising, the mammalian NOX2 enzyme has also been found in endosomes as well as at the plasma membrane; NOX4 has been reported to accumulate in intracellular membranes, the endoplasmic reticulum and even in nuclear compartments (Ushio-Fukai, 2006).

Nowadays, the physiologically controlled production of $O_{2}^{•-}$ by Rboh proteins is known to be implicated in signaling functions related to a variety of processes involved in biotic interactions (plant responses to pathogens and establishment of functional symbiotic nodules) and also related to abiotic stress responses and adaptations (heat, drought, cold, high light intensity, salinity, and wounding); Rbohs also play critical roles in a broad range of developmental functions such as cell growth, both diffuse, and polarized (Marino et al., 2012). Furthermore, Rboh-derived $O_{2}^{•-}$ has been shown to mediate cell-to-cell communication over long distances in plants (Miller et al., 2009).

## The Activity of Plant NADPH Oxidase is Multiregulated

Unlike in animals, NADPH oxidase activity in plants does not require specific interactions with protein partner homologous to the mammal phagocyte protein complex described above. As described below, Rboh proteins possess an N-terminal region involved in specific regulatory mechanisms; this leads signal transduction pathways -namely calcium, protein phosphorylation and lipid signaling- to connect to ROS production (Suzuki et al., 2011).

As with NOX5- and DUOX-type NOX proteins in animals, Rboh proteins display an N-terminal extension with EF-hand motifs (Bedard et al., 2007). Their presence is crucial in Rboh activity, as EF-hands motifs are involved in Ca^2+^-based regulation (Keller et al., 1998) and dimer formation, which has been proposed as the active conformation (Oda et al., 2010). The first crystal structure of the Rboh N-terminal region shows four EF hands, two of which are responsible for dimer stabilization through domain swapping (EF1 of one molecule interacts with EF2 of another molecule and *vice versa*), the remaining two hands being EF-type motifs not predicted from the sequence (Oda et al., 2010). Ca^2+^ binding to EF-hand motifs is essential to trigger oxidative burst, and $O_{2}^{•-}$ production stimulated in this way, in turn, activates Ca^2+^ channels that lead the cation influx into the cell, thus acting as positive feedback (Foreman et al., 2003; Takeda et al., 2008). It is sufficient for Ca^2+^ to bind to only one of the EF hands in order to produce a conformational change in the EF-hand region, which could act as a molecular switch (Ogasawara et al., 2008; Oda et al., 2010). In mammalian NOX5, the binding of Ca^2+^ to EF-hand motifs induces a conformational change that enables the EF-hands to interact with the C-terminus, which is thought to stimulate the enzyme activity (Bánfi et al., 2004). This direct intramolecular interaction between the N- and C-termini also occurs in plant NADPH oxidases, although, unlike in animals, it has been shown to be Ca^2+^ independent and to require the whole N-terminal region (Oda et al., 2010).

In addition to direct Ca^2+^ binding, calcium-regulated protein families, like some calcium-dependent protein kinases (CDPKs) and calcineurin B-like protein-interacting protein kinases (CIPKs), can stimulate Rboh activity via phosphorylation. Two CDPKs from potato were able to induce $O_{2}^{•-}$ production through phosphorylation of two serine residues at the N-terminal extension of StRbohB (Kobayashi et al., 2007; Asai et al., 2013). Using this signaling pathway, Ca^2+^ could act as an oxidative burst inducer through the binding to EF-hands of both Rbohs and CDPKs, which, in turn, phosphorylate, and activate Rboh, ultimately leading to ROS production. In *Arabidopsis*, direct Ca^2+^ binding as well as Ca^2+^-induced phosphorylation by CIPK26 cause a synergistic activation of AtRbohF (Drerup et al., 2013; Kimura et al., 2013). This synergistic effect has also been observed in AtRbohD (Ogasawara et al., 2008), although negative regulation of this isoform by means of a calmodulin-dependent MAPK after wound stress has also been described, suggesting the presence of a feedback pathway to control ROS homeostasis (Takahashi et al., 2011).

Apart from the Ca^2+^-based regulatory mechanism, calcium-independent direct phosphorylation has also been shown. During plant immunity, the plasma-membrane-associated kinase BIK1 (Botrytis-induced kinase1) directly phosphorylates specific residues of RbohD sites in a calcium-independent manner to enhance ROS production (Kadota et al., 2014; Li et al., 2014). Previously, phosphorylation-induced activation had been proposed as a pre-requisite for Ca^2+^-mediated activation, placing phosphorylation at the beginning of the plant Rboh-derived ROS signaling pathway (Kimura et al., 2012). Kadota et al. (2015) have proposed a model integrating Ca^2+^-based regulation and Ca^2+^-independent phosphorylation, in which it is suggested that the latter ‘primes’ RbohD activation by increasing sensitivity to Ca^2+^-based regulation, through Ca^2+^ binding and Ca^2+^-based phosphorylation. The existence of several different kinases acting in sequential or parallel pathways during defense responses has actually been proposed after multiple phosphorylated residues in RbohD were observed (Benschop et al., 2007). The activating effect of Ca^2+^ and phosphorylation on Rboh activity described above could be exerted by increasing the diffusion coefficient, the dimerization state and the clustering in membrane microdomains (Hao et al., 2014).

Small GTPases such as OsRac1 are thought to activate NADPH oxidase activity in order to trigger oxidative burst during plant-pathogen interaction (Ono et al., 2001). Also, Rboh-derived ROS formation in the growing root hair has been shown to depend on the Rop GTPase (Jones et al., 2007), with the receptor-like kinase (RLK) FERONIA (FER) acting as the upstream regulator of the pathway (Duan et al., 2010). A connection with the regulatory mechanism described above is suggested in the model proposed by Wong et al. (2007), where Ca^2+^-dependent phosphorylation leads to a conformational change in Rbohs that facilitates Rac binding. This effect could subsequently be suppressed by means of ROS-induced Ca^2+^ accumulation, which would act as negative feedback of NAPH oxidase activity. The Rac-Rboh interaction takes place in the coiled-coil region created by EF-hand swapping, with dimers being suggested to be the functional units for the binding (Oda et al., 2010).

Zhang and co-authors have shown that phosphatidic acid (PA) produced by phospholipase Dα1 (PLDα1) interacts with the N-terminal part of AtRbohD during the ABA response, thus activating ROS production with the downstream involvement of nitric oxide (NO) (Zhang et al., 2009). NO had previously been reported to be linked to ROS at several levels (Delledonne et al., 2001; Delledonne, 2005) and to act as a negative regulator of NADPH oxidase through S-nitrosylation of a conserved Cys in the C-terminal part, which has been suggested to be a conserved mechanism to regulate immune responses in both plants and animals (Yun et al., 2011).

Other less documented interactions include the induction of NADPH oxidase-dependent superoxide production in *Arabidopsis* leaves by extracellular ATP, with cytosolic Ca^2+^ also probably being involved in this signaling network (Song et al., 2006). Finally, RbohD isoform activity has recently been linked to vesicle trafficking. Thus, clathrin-dependent pathways as well as the presence of membrane microdomains affect protein endocytosis, which changes the amount and mobility of the protein at the plasma membrane (Hao et al., 2014).

## NADPH Oxidase Activity During Development of the Female Gametophyte

**FIGURE 1:**
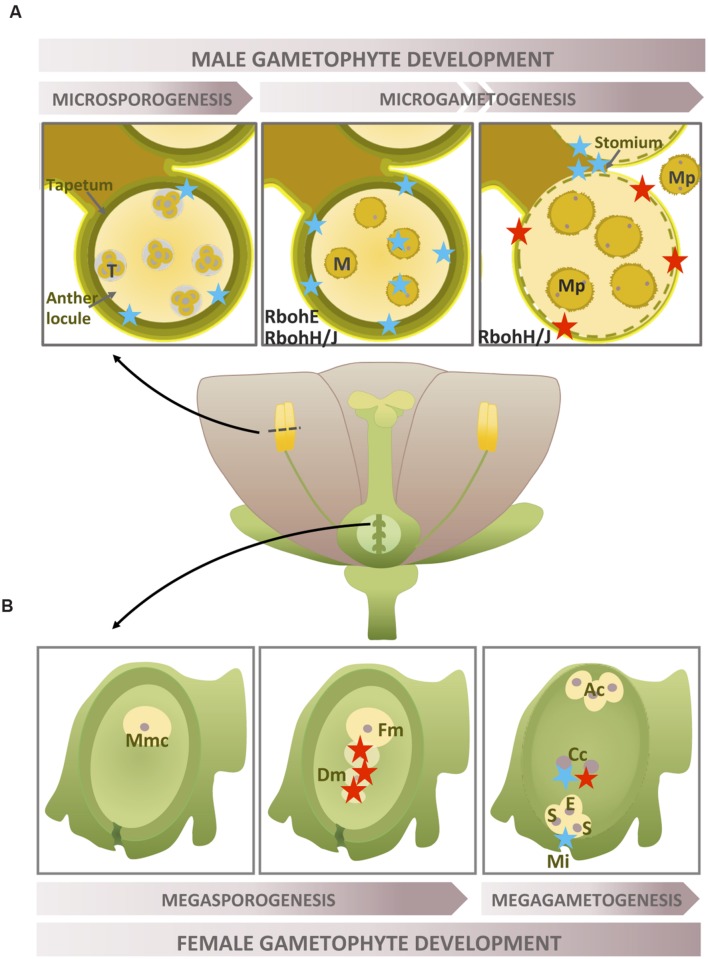
**Rboh-dependent superoxide production during gametophyte development.** Location of $O_{2}^{•-}$ during the development of gametophytes. **(A)** Two selected stages of late anther development (tetrads and microspores) and anthers at the dehiscence stage. **(B)** Three selected female gametophyte development stages, including the pre-meiotic phase, functional megaspore formation and the fully developed embryo sac stage. The localization of H_2_O_2_ accumulation is also shown when necessary (for more detailed information, see text above). Recognized sources of superoxide are marked in black. Blue star: $O_{2}^{•-}$; red star: H_2_O_2_; T, tetrads; M, microspore; Mp, mature pollen; Mmc, megaspore mother cell; Fm, functional megaspore; Dm, degenerating megaspore; Ac, antipodal cell; Cc, central cell; S, synergid; E, egg cell; Mi, micropyle.

In angiosperms, the female gametophyte or megagametophyte, is the embryo sac originating from a haploid megaspore. During gametogenesis, mitochondria have been established as the primary source of mainly superoxide and hydrogen peroxide (Martin et al., 2014a). Although ROS have been shown to be not just involved in but also tightly regulated in megagametogenesis (Martin et al., 2013, 2014b), little information on ROS/superoxide during female gametophyte development is available. These studies analyze female gametophytic mutants impaired in MnSOD activity which show infertility caused by various defects ranging from development arrest to aberrant egg apparatus. In WT ovules, ROS (H_2_O_2_) were detected early on in the process, when megaspore cell death takes place (**Figure 1B**). At a later stage of development, the mature female gametophyte showed both mitochondrial superoxide and peroxide accumulation in the central cell, whereas cytosolic superoxide was only detected outside the embryo sac at the micropylar portion (**Figure 1B**). Alternatively, as the mutant ovules showed abnormally high levels of ROS, including cytosolic superoxide, the authors suggest that other sources of ROS, such as NADPH oxidase, might also be involved. Transcriptome data concerning ovule development have demonstrated that *RbohD* shows the highest expression, whereas other Rboh isoforms display quite low expression levels (Wynn et al., 2011). To our knowledge, no female gametophyte-specific Rbohs have been described to date.

## NADPH Oxidase Activity During Development of the Male Gametophyte

Superoxide/ROS production during pollen ontogeny has been little studied. Superoxide was detected during rice anther development (Hu et al., 2011) and showed a stage-dependent type of production; it peaked when the formation of young microspores took place and exhibited low levels during the remaining stages and increased slightly when pollen achieved maturation (**Figure 1A**). The subcellular location of $O_{2}^{•-}$ was determined in the tapetal cells and microspores and may be associated with the initiation of tapetal programmed cell death (PCD). The tapetum is the innermost sporohytic layer in the anther and undergoes cell degeneration to support pollen development after meiosis (Wu and Cheun, 2000). In olive flowers, anthers did not show large amounts of superoxide until the dehiscence stage, when it was localized in the stomium. At this developmental stage, massive production of superoxide together with other ROS (mainly H_2_O_2_) could also be involved in PCD mechanisms, affecting the endothecium and the surrounding connective tissues as well as the stomium (Zafra et al., 2010). Few data are available on the origin of anther superoxide apart from the involvement of RbohE in the tapetal PCD in *Arabidopsis* anthers (Xie et al., 2014). Transcriptome data concerning microgametogenesis analyzed from the haploid microspore to the mature functional pollen in *Arabidopsis* showed that *RbohH/J* are highly expressed in both the immature and mature tricellular pollen (Honys and Twell, 2004).

## Putative Roles of NADPH Oxidases During Pollen-Stigma Interaction and Pollen Tube Growth Through the Female Tissues

### Pollination

**FIGURE 2:**
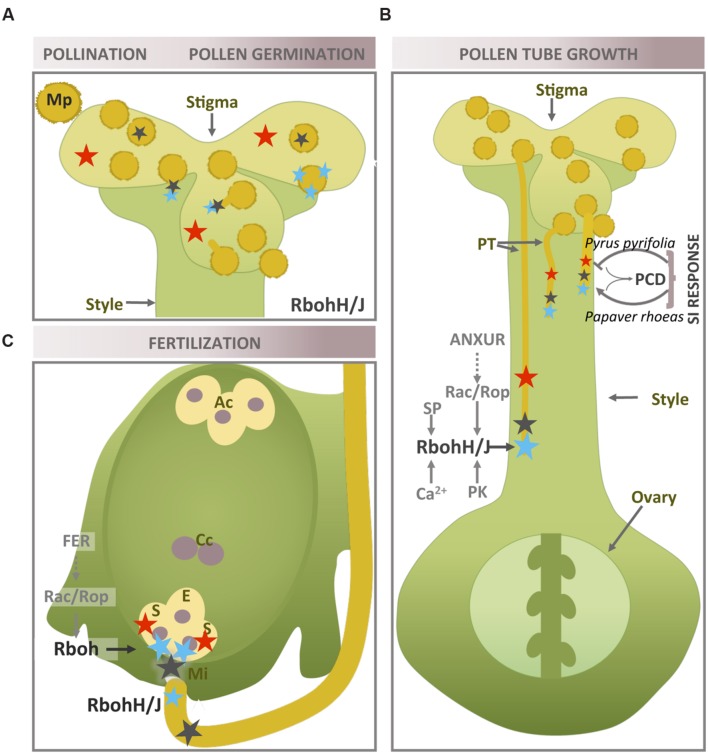
**Rboh-dependent superoxide production in plant reproductive tissues during pollen–pistil interaction.** Location of $O_{2}^{•-}$ in reproductive tissues during pollination and pollen germination **(A)**, pollen tube growth **(B)** and fertilization **(C)**. The location of H_2_O_2_ and NO is also shown when necessary (more information in text above). Recognized superoxide sources and regulatory elements are marked in black. Blue star: $O_{2}^{•-}$; red star: H_2_O_2_; black triangle: NO; PT, pollen tube; Ac, antipodal cells; Cc, central cell; S, synergid; E, egg cell; PK, protein kinase; SP, signaling phospholipid; Mi, micropyle.

The constitutive accumulation of H_2_O_2_ at the stigmatic papillae when receptivity peaks has been reported in a large number of angiosperms (**Figure 2A**), and the presence of potential crosstalk with pollen-generated NO has been suggested (McInnis et al., 2006; Zafra et al., 2010). However, the enzymatic source of this H_2_O_2_ remains unknown. Recently, ROS accumulation upon pollination has been shown to occur in a Rboh-dependent manner in the apoplast/cell wall of the pollen tube touching the stigmatic papillae, since *Arabidopsis rbohH rbohJ* double mutants lacking NADPH oxidase activity are deficient in this feature (Kaya et al., 2015). These authors have proposed that Rboh-generated ROS could assist pollen tube elongation by facilitating the generation of a flexible cell wall tip.

On the other hand, production of cytosolic superoxide as well as peroxide, described following pollination in the *Arabidopsis* embryo sac, is restricted to the synergid cells and is maintained until the pollen tube arrives (Martin et al., 2013, 2014b). The authors believe that the presence of ROS depends on pollination, suggesting that the female gametophyte is able to sense a signal preceding pollen tube contact, although no information on the superoxide source involved is provided. Furthermore, analysis of transcriptomic data during pollen–pistil interactions once again showed that RbohD is the highest expressed isoform in the pistil (Boavida et al., 2011).

### Onset of Pollen Germination

Just before the pollen tube emerges, the cytoplasm of the hydrated mature pollen undergoes major changes such as cytoskeleton reorganization, vesicle accumulation near the aperture where pollen tube emerges as well as local thinning of the intine. Apart from these structural alterations, there is also evidence of changes in the presence and localization of ROS (**Figure 2A**). Higher concentrations of ROS are produced in both the cytoplasm and cell wall of the pollen grains at the very beginning of the germination process (Smirnova et al., 2014). These extracellular ROS, specifically H_2_O_2_, are known to be involved in pollen grain activation (Speranza et al., 2012). Among the ROS involved in pollen germination, $O_{2}^{•-}$ was detected following pollen rehydration and later at the germination aperture. This occurrence of $O_{2}^{•-}$ is prevented by the NADPH oxidase inhibitor DPI, which also reduces pollen germination in tobacco and kiwi fruit (Potocky et al., 2012; Speranza et al., 2012).

Allergenic pollen grains have been reported to contain NADPH oxidase activity that differed in intensity and localization according to the plant families studied. Thus, the activity was described at the pollen surface and in the cytoplasm, in subpollen particles released from pollen and at the inner pollen surface, and, in all cases, was mostly concentrated in insoluble fractions (Wang et al., 2009). The presence of this enzyme in numerous allergenic species led the authors to suggest that there is a possible link between NADPH oxidase activity/ROS and allergy, which has been further tested by other authors (Bacsi et al., 2005; Boldogh et al., 2005). However, the findings available in the literature on this issue are contradictory (Shalaby et al., 2013).

After the pollen tube emerges, NADPH oxidase-produced $O_{2}^{•-}$ appears only in the growing tip and has been proposed as a key element in cell polar expansion (Potocky et al., 2012). This hypothesis has been discussed by others authors, who suggest that superoxide influences pollen germination only indirectly, as it can be spontaneously transformed into H_2_O_2_, which subsequently, together with OH, regulates tobacco pollen germination by affecting the mechanical properties of the intine (Smirnova et al., 2014). It is important to note that some discrepancies have been reported regarding the role played by NADPH oxidase activity in certain species, such as cucumber, in which DPI and catalase were found to slightly promote pollen germination instead (Sirova et al., 2011).

### Pollen Tube Growth

Tip growth consists of elongation exclusively at the apex through the polarized exocytosis of the membrane’s newly synthesized components to the apical elongation domain. Furthermore, tip-growing cells, such as pollen tubes, or root hairs, are sensitive to pistil and soil environments, thus making signaling an important feature in these kinds of cells (Feijo et al., 2004). Several molecules, including ROS and Ca^2+^, have been reported to be involved in apical growth (Coelho et al., 2008). It is important to consider the role of other ions, such as potassium, the effect of pH, lipid signaling and small GTPases in the context of cytoskeletal reorganization and polar vesicle trafficking (Feijo et al., 2004; Cole and Fowler, 2006; Lee and Yang, 2008) as well as NO and cGMP (Prado and Feijó, 2009).

NADPH oxidase activity is involved in apical growth in roots, pollen tubes and in the polarized zygotic growth of the model alga *Fucus serratus* (Foreman et al., 2003; Potocky et al., 2007; Coelho et al., 2008). In root hair cells, NADPH oxidase activity is located at the growing tip during elongation, causing superoxide accumulation in the cell wall which facilitates rapid elongation (Dunand and Penel, 2007) and disappearing when growth stops (Takeda et al., 2008). Rboh mediates cell elongation through the production of local ROS waves at the root hair tip (Monshausen et al., 2007) which, in turn, could activate Ca^2+^ channels (Foreman et al., 2003).

In *Arabidopsis thaliana*, two genes *RbohH* and *RbohJ* are specifically expressed in the pollen grain and the growing pollen tube (Sagi and Fluhr, 2006; Kaya et al., 2014). Initial approaches to the study of pollen-specific Rbohs linked the presence of superoxide at the pollen tube apex to the activity of these enzymes, as this process was affected by transfection with specific *Rboh*-antisense oligodeoxynucleotides (ODNs) and by DPI. These treatments also inhibited pollen tube growth, leading to the conclusion that superoxide production by NADPH oxidase activity is essential for apical elongation. Moreover, as previously observed for other isoforms, pollen NADPH oxidase activity is stimulated by calcium (Potocky et al., 2007; Speranza et al., 2012). However, a recent study has suggested that the decrease in DPI-mediated growth could be due to the inhibition of other flavoenzymes in addition to Rbohs (Lassig et al., 2014). In fact, mitochondria were also proposed as the source of these ROS in *Lilium formosanum* (Cardenas et al., 2006). In this pollen, mitochondrial NAD(P)H dehydrogenase activity correlates with the oscillation of the growth rate. These authors observed the highest ROS production at the subapical region, where most mitochondria are located, rather than at the tip. Moreover, in the cucumber pollen tube, both these localizations occurred at different times: although ROS and NO were detected at the tip at the beginning of tube growth process, their presence then extended to the whole tube (Sirova et al., 2011). In this line of argument, superoxide production was detected in kiwi fruit pollen with no clear localization at the tip (Speranza et al., 2012). To reconcile these contrasting views, other authors have suggested the presence of two ROS sources in growing pollen tubes (**Figure 2B**): NADPH oxidase at the very tip and mitochondria in the subapical region (Liu et al., 2009). Nevertheless, a recent study has shown that the pollen-specific Rbohs, RbohH and RbohJ, from *A. thaliana*, show overlapping localization up to a point: they both appeared in the subapical region very close to the tip, although RbohJ is the only isoform present in the pollen shank (Lassig et al., 2014).

With regard to subcellular localization, initial analyses carried out using gel blotting and determination of cell fraction activity (Potocky et al., 2012) followed by further experiments with fusion proteins (Boisson-Dernier et al., 2013; Kaya et al., 2014) have shown that pollen Rboh isoforms are situated at the plasma membrane of the pollen tube. Targeting depends on endosomal recycling, with RbohH and RbohJ showing differences in internalization kinetics (Lassig et al., 2014). With respect to the pollen plasma membrane, it has been suggested that Rboh proteins are partially included in specific lipid microdomains in *Picea meyeri* (Liu et al., 2009). In addition, lipid microdomain polarization at the tip during pollen tube growth was shown to be necessary for NADPH oxidase activity, the establishment of a calcium gradient and subsequent apical expansion. Curiously, although they would be expected to be situated in the plasma membrane only, RbohH and RbohJ are actually also localized at the cytoplasm (Lassig et al., 2014).

### Controlling Pollen Tube Growth Rate and Cell Wall Integrity

In growing pollen tubes, ROS produced by NADPH oxidase activity have been shown to regulate the balance between cell wall extensibility and strength, this structure’s two main cytomechanic properties (Carol and Dolan, 2006). ROS production could be involved in two counteracting processes: loosening of the cell wall and cross-linking of cell wall components (Smirnova et al., 2014). In fact, the critical role played by ROS in the cytomechanic characteristics of the cell wall has also been demonstrated in anther tissues. In these tissues, the chloroplast redox system, comprised of proteins containing the cystathionine β-synthase domain CBSX, thioredoxins and peroxiredoxins, is able to connect plant nutritional information and pollen release by controlling the extracellular level of hydrogen peroxide during anther dehiscence (Ok et al., 2012; Jung et al., 2013).

A recent study investigates the *rbohH rbohJ* double mutant, which shows unstable growth as compared to the low fluctuation rates found in WT lines (Lassig et al., 2014). According to this study, two growth patterns were established in this double mutant: (a) periods with short bursts of growth followed by growth cessation and (b) periods with elevated average growth rates, which eventually culminated in pollen tube collapse. The authors of the study established a pivotal role for RbohH and RbohJ, which involves modulating growth rate oscillations in order to facilitate coordination with exocytosis. This original proposal is based on the fact that the double mutant undergoes thinning of the apical cell wall due to the lack of building material, which ultimately forces the pollen tubes to stop growth. This is followed by a thickening of the cell wall owing to apical cell wall deposition or even a collapse due to the excessive increase in exocytosis. NADPH oxidase-produced ROS appears to act as a speed regulator presumably by rigidifying the cell apex. Apart from mediating in cell elongation and shaping the pollen tubes, it has been suggested that pollen apoplastic ROS mediate cell wall loosening and facilitate pollen tube growth through female tissues (Kaya et al., 2015).

The importance of pollen Rbohs in maintaining cell wall integrity during tube growth is also highlighted in a study (Boisson-Dernier et al., 2013) in which it is reported that over-expression of *RbohH* and *RbohJ* causes over-activated exocytosis and accumulation of secreted membrane. This results in aberrant tube morphology, thus suggesting that RbohH/J activity should be tightly regulated.

## Regulating NADPH Oxidase Activity in Pollen

Many of the aforementioned regulatory mechanisms present in vegetative Rbohs caused by physiological or stress conditions have also been described for pollen Rbohs (**Table 1**).

**Table 1:** Rboh multiregulation in vegetative tissues vs. the pollen tube.

	Vegetative tissues	Pollen tube	Modulation
Ca^2+^	Direct binding to EF hands motifs Indirectly: Ca^2+^-induced phosphorylation; binding to Rop-GTPase	Synergistic activation

Phosphorylation	CPK5 CIPK26 MAPK cascades	Unidentified candidates (pollen tube CPK proposed)	Synergistic activation

Small GTPases	Rac1 Rop2	Rop1 Rac5	Activation Targeting?

Low abundance of signaling phospholipids	PA produced by PLDα1	PA (synergism with Ca2+) PIP2	Activation

NO	S-nitrosylation	Putative, not described	Negative

The second messenger Ca^2+^ and several Ca^2+^-associated proteins are well-known key elements involved in pollen tube growth, as a tip-focused Ca^2+^ gradient as well as oscillations in intracellular concentrations are required (Cole and Fowler, 2006; Cardenas et al., 2008). As mentioned above, plant Rbohs contain two Ca^2+^-binding EF-hand motifs in the cytoplasmic portion. In an initial approach, extracellular calcium was used to increase/activate pollen NADPH oxidase activity *in vitro* and *in vivo*, which was observed to have a dose-dependent effect (Potocky et al., 2012). Kaya et al. (2014) went further and, by means of the transitory expression of *RbohH* and *RbohJ* and the induction of Ca^2+^ influx, showed that both proteins effectively displayed Ca^2+^-activated NADPH oxidase activity. Once again, it was demonstrated that the presence of EF-hands motifs is essential for this activity, as the mutation in two critical positions for Ca^2+^ quelation in the binding loop leads to impaired Ca^2+^-induced NADPH oxidase activity. The authors also suggest that, as with RbohD, the conformational change in the second EF-hand region is required. In turn, pollen Rbohs affect Ca^2+^ dynamics in the growing tubes; in the *Arabidopsis rbohH rbohJ* double mutant, the tip-focused Ca^2+^ gradient was destabilized and reduced rather than eliminated. Furthermore, an increase in external Ca^2+^ partially rescued the phenotype (Boisson-Dernier et al., 2013). In this study, the authors suggest that pollen Rbohs are not required to generate a Ca^2+^ gradient, which, however, is adjusted and stabilized to support regular growth, as suggested previously with respect to RbohC in *Arabidopsis* root (Monshausen et al., 2007). Moreover, Lassig et al. (2014) report that, in this double mutant, the steady tip-focused gradient is replaced by intracellular Ca^2+^ bursts preceded by short growth peaks followed by growth cessation. Accordingly, they propose that NADPH oxidase activity is an indirect modulator of intracellular Ca^2+^ dynamics, although which factor operates upstream seems to be unclear. Positive feedback appears to occur during pollen tube growth, with NADPH oxidase activity depending on Ca^2+^ and the ROS produced then maintaining the tip-focused Ca^2+^ gradient. In fact, as in the case of *Arabidopsis* root hairs, ROS-activation of Ca^2+^ permeable channels in the plasma membrane of pollen tubes has been detected (Wu et al., 2010).

Synergistic activation of NADPH oxidase activity by Ca^2+^ and phosphorylation has also been reported (Kaya et al., 2014), although RbohH/J activation through phosphorylation lacks identifiable candidates. Although several CDPKs involved in pollen tube growth could be proposed, current models are based on experimental data from Rboh isoforms that differ from those present in pollen (Wudick and Feijo, 2014). Similarly, the low-abundant signaling phospholipids PA and phosphatidylinositol 4,5 biphosphate (PIP_2_) were found to promote NADPH oxidase activity both *in vitro* and *in vivo*, (probably in a synergistic way with calcium), and PA is also likely to play a role downstream of NADPH oxidase activity. In a similar set of experiments with tobacco pollen, small GTPases from the Rop family were shown to act as pollen Rbohs regulators *in vivo*. Moreover, protein targeting to the pollen tube tip could be affected by Rop GTPases (Potocky et al., 2012), although the relationship between Rop and Rboh proteins needs further investigation (Kaya et al., 2014).

Moreover, the pollen ANXUR RLKs, located at the tip-growing tube, have been placed upstream of RbohH and RbohJ and acts as a regulatory element in the pollen tube growth (Boisson-Dernier et al., 2013). As discussed above, Rbohs from root hairs are known to be activated by RLKs through Rop signaling (Duan et al., 2010), although this pathway has not yet been observed in pollen tubes (Boisson-Dernier et al., 2013). Recently, Wudick and Feijo (2014) have developed a clarifying model of ROS signaling in the pollen tube showing the positive feedback discussed above. In this model, small GTPases together with RLKs are considered to be promoters of ROS-production. They describe an initial phosphorylation step, which facilitates Ca^2+^ binding to pollen Rbohs and subsequent ROS production, which, in turn, could activate Ca^2+^ channels. The increase in calcium could, in turn, activate some CDPKs, with a consequent intensification of Rboh phosphorylation and ROS production. Downstream of RbohH/J, a receptor-like cytoplasmic kinase (RLCK), named MARIS (MRI) expressed preferentially in pollen tubes and root hairs, has recently been added to this signaling cascade which seems to ultimately control cell wall integrity during pollen tube tip growth (Boisson-Dernier et al., 2015).

## Self-Incompatibility (SI) Response to NADPH Oxidase Activity

The self-incompatibility (SI) response consists of a collection of molecular and cellular mechanisms capable of preventing self-fertilization by suppressing pollen germination and tube growth. Redox signaling by reactive species (ROS/NO) has been shown to be clearly involved in these processes. SI is based on stigma and pollen recognition, with three main SI models driven by different genes having been proposed, all three of which use a multiallelic S-locus system (Takayama and Isogai, 2005). The combination of different haplotypes enables discrimination between compatible and incompatible interaction- rejection of incompatible pollen occurs through different mechanisms, including PCD, which is ultimately triggered in incompatible pollen tubes. ROS, together with NO, are key actors in the course of PCD in plants (Wang et al., 2013), and their involvement in the SI response is highlighted in **Figure 2B**. However, as SI systems and S-determinants have yet to be determined in many plant families (Dresselhaus and Franklin-Tong, 2013), these signaling mechanisms require further study (Serrano et al., 2015).

Pear (*Pyrus pyrifolia* L.) shows an S-RNase-based gametophytic SI mechanism, consisting of a specific inhibition of self-pollen germination and tube growth by the style S-RNase. As already discussed above, tip-localized ROS are necessary for pollen tube growth; nevertheless, the pollen tubes of incompatible pear pollen showed a disruption in NADPH oxidase- and mitochondria-mediated ROS production (Wang et al., 2010). The authors show how this SI-mediated ROS disruption elicits a decrease in Ca^2+^ as well as actin cytoskeleton depolymerization and nuclear DNA degradation - the latter two processes being considered key markers of PCD (Wang et al., 2013) – and how DPI and a ROS scavenger are used to support the involvement of Rboh and ROS. Interestingly, an extracellular apoplastic calmodulin (CaM) from the transmitting tissues of the pistil managed to rescue self-pollen tube growth *in vivo* and *in vitro*, possibly through the induction by Ca^2+^ of current and subsequent generation of tip-localized superoxide/ROS and through stabilization of actin filaments. In addition, CaM- dependent stabilization of actin filaments could occur through cross-talk between cytosolic free calcium and ROS (Jiang et al., 2014). However, direct evidence of Rboh involvement has not been obtained.

*Papaver rhoeas* displays a different SI mechanism in which the S-locus includes pollen and pistil S-determinants, whose interaction in an incompatible combination results in the rapid inhibition of pollen tube tip growth involving PCD. This mechanism initially involves a Ca^2+^-dependent network, which leads to a relatively rapid and temporary increase in the levels of ROS and NO (Wilkins et al., 2011). Both reactive species act upstream of SI markers such as DEVD/caspase-3-like activity and actin cytoskeleton reorganization, suggesting that ROS and NO together activate PCD in the SI response. The sharp rise in ROS is diffusely localized in the tube shank and in indeterminate spots, whose origin is controversial, as the authors propose sources different, at least in part, from the NADPH oxidase. In olive trees (*Olea europaea* L.), PCD has been involved in the rejection of incompatible pollen by arresting its growth in the style. During *in vitro* pollen germination, the increase in tip-localized superoxide in the course of self-incompatible experiments was accompanied by an increase in the gene expression of some Rboh isoforms (Serrano et al., 2012). The authors suggest that superoxide as well as NO are key signaling molecules in the interaction between the incompatible pollen and the pistil, which can trigger PCD programs (**Figure 2B**).

With regard to the other SI model for *Brassicaceae*, no clear involvement of ROS/NO has yet been described. Nevertheless, in this model, the different molecular mechanisms involved in the redox signaling role played by thiol-based redox protein thioredoxins have been highlighted and outlined (Cabrillac et al., 2001; Ivanov and Gaude, 2009).

## Fertilization and Superoxide Production

The ultimate goal of pollen tube growth is to ensure that the female gametophyte delivers the sperm nuclei. Once again, the characterization of the two *Arabidopsis Rboh* genes specifically expressed in pollen, using the respective single and double mutant, shows the important role played by these genes in the fertilization process. Self-fertilization rates in the single *rbohH* and *rbohJ* mutants were comparable to wild-type rates (Kaya et al., 2014). However, the double mutant, which was partially sterile, was rarely found in the progeny (Boisson-Dernier et al., 2013) and had shorter siliques and fewer seeds than WT (Boisson-Dernier et al., 2013; Kaya et al., 2014; Lassig et al., 2014). This is explained by the mutant pollen’s inability to reach the female gametophyte *in vivo*, while most of the pollen tubes were actually found to break up during *in vitro* germination. In other words, the disruption of RbohH and RbohJ prevented the fertilization of the female gametophyte. Boisson-Dernier et al. (2013) have suggested that the functional redundancy of RbohH and RbohJ is partial, with RbohH being able to compensate for the loss of RbohJ, although this substitution was only partial in the opposite direction. Moreover, the authors observed tube rupture during *in vitro* germination in the *rbohH* single mutant, although this phenotype did not significantly reduce seed production. NO has also been shown to be involved in ovule micropyle addressing (**Figure 2C**) (Prado et al., 2008). RbohH and RbohJ could be putatively involved in ovule targeting, as both proteins carry the conserved Cys residue, which is effectively nitrosylated in RbohD (Yun et al., 2011). However, this post-translational modification has still not been described in pollen.

As ROS are detected in the synergid cells upon the arrival of the pollen tube (Martin et al., 2014a), NADPH oxidase activity could be involved in plant fertilization through another mechanism. Once pollen tube has entered the embryo sac via the micropyle, the female gametophyte induces its rupture, and the sperm cells are released and made available for fertilization. A recent study has shown that female gametophyte NADPH oxidase-dependent ROS are necessary for this ovule task to succeed (Duan et al., 2014). ROS generated at the entrance of the embryo sac (filiform apparatus/synergid cell region) reach a maximum level, coinciding with maximum ovule receptivity; the ROS cause both this tube rupture, which is Ca^2+^-dependent *in vivo* and *in vitro*, and sperm release. Thus, the application of ROS to *Arabidopsis* pollen tubes leads to a sharp increase in Ca^2+^ in the distal cytoplasm, immediately followed by pollen tube rupture, suggesting the presence of Ca^2+^ -signaling events downstream in the pollen tube. As suggested in relation to root hairs, the FERONIA receptor-like kinase (FER) acts upstream of Rop. FER is broadly expressed except in pollen, where, as previously mentioned, the pollen-specific ANXUR homologs are upstream of RbohH and RbohJ, (**Figures 2B,C**) (Boisson-Dernier et al., 2013).

After fertilization, the exclusion of ROS from the fertilized embryo sac is required for the embryo to develop properly, although no biological significance has been proposed in relation to the remaining superoxide at the micropylar end of the integuments (Martin et al., 2013, 2014b).

## Conclusion

In this study, we review evidence on the critical involvement of ROS generated by plant NADPH oxidases in a broad range of processes related to sexual plant reproduction. From anther development to pollen germination and pollen tube elongation through polarized growth until fertilization, several plant Rboh isozymes have been identified as specifically expressed in the male gametophyte, where they play important reproductive roles under tightly regulated conditions. Although the identified forms of Rboh appear to show at least partially redundant functions, some questions remain in relation to the regulation of RbohH/J in the pollen tube. For example, the positive feedback mediated by Ca^2+^ appears to occur in pollen, where, as occurs with roots hairs, RbohH/J-produced ROS may activate as yet unidentified plasma membrane Ca^2+^ permeable channels. This positive feedback is probably a conserved mechanism at the apex of tip-growing cells, which could be involved in maintaining polarity. Such an important regulatory mechanism as phosphorylation in Rboh isoforms continues to constitutes a gap in the RbohH/J model, as phosphorylation agents (Ca^2+^-dependent and non-Ca^2+^-dependent) have not yet been identified. Consistent with the findings on vegetative tissues, small GTPases from the Rop/RAC family affect Rboh activity in pollen tubes, although further studies are necessary in order to investigate this interaction and its putative effect on RbohH/J localization. In root hairs, FER activates RbohC-dependent ROS production through ROP2 signaling, whereas, in pollen tubes, ROP involvement remains unclear. Apical growth models for root hair and pollen tubes, for example, have been shown to share common features, although the dissimilar physiological functions, cell wall characteristics and growth environments need to be taken into account.

Several SI mechanisms depend on ROS pathways, and successful fertilization is likely to be achieved through Rboh isoforms capable of affecting pollen tube dynamics. These data also suggest that Rbohs are strongly involved in crosstalk between pollen and pistil. However, considerably less information has been collected on the generation of superoxide produced by NADPH oxidases in the gynoecium. Thus, unidentified female gametophyte-expressed Rboh(s) generate(s) ROS that can induce tube burst and sperm cell delivery, probably by means of cell wall weakening. A distinction needs to be drawn between this specialized mechanism and premature abnormal pollen tube rupture caused by the *rboh* mutant, which could be due to a previous increase in the rate of exocytosis whose cause is unknown. The integration of signaling pathways and known regulators could lead to the development of a model in which many remaining issues might be resolved. Moreover, despite the recent advances made, apart from the *Arabidopsis* model, little information is available regarding Rboh proteins in the reproductive tissues of plants. NADPH oxidase-dependent superoxide production in the reproductive tissues of species of agronomic value should be an important subject of study in the future.

## Author Contributions

MJ-Q, JT, and JA designed the review, collected the literature available and wrote the manuscript. All authors reviewed and approved the manuscript.

## Conflict of Interest Statement

The authors declare that the research was conducted in the absence of any commercial or financial relationships that could be construed as a potential conflict of interest.
